# Coccidioidomycosis-associated Hospitalizations, California, USA, 2000–2011

**DOI:** 10.3201/eid1910.130427

**Published:** 2013-10

**Authors:** Gail Sondermeyer, Lauren Lee, Debra Gilliss, Farzaneh Tabnak, Duc Vugia

**Affiliations:** California Department of Public Health, Richmond, CA, USA

**Keywords:** coccidioidomycosis, Coccidioides, fungal, hospitalizations, California, public health, pneumonia, meningitis, fungi, epidemiology, Valley fever

## Abstract

In the past decade, state-specific increases in the number of reported cases of coccidioidomycosis have been observed in areas of California and Arizona where the disease is endemic. Although most coccidioidomycosis is asymptomatic or mild, infection can lead to severe pulmonary or disseminated disease requiring hospitalization and costly disease management. To determine the epidemiology of cases and toll of coccidioidomycosis-associated hospitalizations in California, we reviewed hospital discharge data for 2000–2011. During this period, there were 25,217 coccidioidomycosis-associated hospitalizations for 15,747 patients and >$2 billion US in total hospital charges. Annual initial hospitalization rates increased from 2.3 initial hospitalizations/100,000 population in 2000 to 5.0 initial hospitalizations/100,000 population in 2011. During this period, initial hospitalization rates were higher for men than women, African Americans and Hispanics than Whites, and older persons than younger persons. In California, the increasing health- and cost-related effects of coccidioidomycosis-associated hospitalizations are a major public health challenge.

Coccidioidomycosis, also known as Valley fever, is a reemerging infectious disease caused by inhalation of *Coccidioides* fungi spores, which reside in the soil of regions where coccidioidomycosis is endemic, including the southwestern United States (*1*–*5*). California and Arizona have the highest rates of reported coccidioidomycosis cases in the United States (*4*,*6*). In California, the pathogen is most common in the San Joaquin Valley, and compared with other Californians, residents of this region are at increased risk for infection (*6*,*7*). Although most coccidioidomycosis cases are asymptomatic, symptomatic disease will develop in ≈40% of patients and can range from self-limited influenza-like illness to disseminated disease and chronic meningitis (*7*,*8*). Symptomatic disease can require expensive and aggressive treatments, prolonged absence from work or school, multiple hospitalizations, and years of monitoring (*9*,*10*). Populations at particular risk for severe disease include African Americans, immunocompromised persons, and persons >65 years of age (*2*,*11*).

Over the past decade, increases in the number of reported cases of coccidioidomycosis have been documented from Arizona and California (*6*,*12*–*16*). In California, the rates of reported cases increased >6-fold from 2000 to 2011 (2.4 to 14.4 cases/100,000 population, respectively) (*13*,*15*). Cases among prisoners in California also increased during this time, making prisoners a population of concern (*17*,*18*). To determine the epidemiology, extent, and effect of the disease in California, we reviewed coccidioidomycosis-associated hospitalizations in the state for 2000–2011.

## Methods

### Data Source

We used the California Patient Discharge Data Set, an administrative database developed by the Office of Statewide Health Planning and Development, to review hospitalization data for 2000–2011 (*19*). The database contains inpatient (defined as a person formally admitted to the hospital with the expectation of remaining overnight or longer) information reported by nonfederal hospitals in California (*20*). These data include diagnosis and procedure codes, demographic characteristics, hospital admission and discharge dates, source of admission, and hospital charges. Records for coccidioidomycosis-associated hospitalizations were identified as records with an admission date during January 1, 2000–December 31, 2011, and 1 of the following primary or secondary International Classification of Diseases, 9th revision (ICD-9) codes for coccidioidomycosis: 114.0 primary pulmonary, 114.1 primary extrapulmonary, 114.2 meningitis, 114.3 other forms of progressive, 114.4 chronic pulmonary, 114.5 pulmonary unspecified, and 114.9 unspecified.

Hospitalization records were matched by patients’ social security numbers, dates of birth, sex, race/ethnicity, and county of residence by using probabilistic record linkage methods as described (*21*,*22*). Matching of records by patient enabled the classification of all hospitalization records into initial and subsequent hospitalizations for each patient. Records missing the social security number or other data elements were included in the record linkage and subsequent analyses.

### Hospitalization Rates

Numbers of coccidioidomycosis-associated hospitalizations were calculated for each year during 2000–2011, by initial and subsequent hospitalization, and by type of coccidioidomycosis. Hospitalization rates were calculated per 100,000 population by using population estimates from the California Department of Finance that were based on the 2010 census (*23*,*24*).

### Demographic Characteristics

Subanalyses were performed for patient initial hospitalizations by sex, age group, race/ethnicity, and county and region of patient residence. Two regions of California were defined for this study: areas where coccidioidomycosis is endemic (hereafter referred to as the endemic region) and areas where coccidioidomycosis is less endemic (hereafter referred to as the less endemic region). The endemic region was defined as the 6 counties that had annual case rates consistently higher than those for the state during 2001–2011: Fresno, Kings, Kern, Madera, San Luis Obispo, and Tulare Counties (*6*,*12*–*14*). The less endemic region was defined as all other California counties. Descriptive statistics were also calculated for hospital admissions from prisons or jails.

### Concurrent Conditions

We reviewed diagnosis codes for the initial hospitalization records to identify patients with the following concurrent conditions: immunocompromised state as defined by the Agency for Healthcare Research and Quality (*25*), HIV and AIDS (ICD-9 codes 042, V08), diabetes (ICD-9 code 250), and pregnant state (ICD-9 codes V22, V23, 630–679). The estimated percentage of the California population with these conditions was then provided as a reference. The estimated percentage of the California population with HIV infection or AIDS was determined by using data from the California Department of Public Health HIV/AIDS Surveillance Statistical Reports for 2004–2011, and the estimated percentages of the California population with diabetes and with pregnancy were determined by averaging data from the California Health Interview Survey (CHIS) for 2003, 2005, 2007, and 2009 (*26*,*27*). For diabetes, both the percentage in our study and the estimated population average were age-adjusted to the 2000 standard US population. We were unable to determine the prevalence of non-HIV immunocompromised state in the population.

###  Length of Stay and Hospital Charges

Length of stay was calculated for all initial and subsequent hospitalizations and was defined as the number of days from admission to discharge for each hospitalization. Total length of stay per person was defined as the sum of the lengths of stay for an individual patient’s initial and subsequent hospitalizations during the study period.

Total hospital charge per patient was calculated by adding the total charges for all hospitalizations during 2000–2011. Total hospital charge per day was determined by dividing the total charge for each hospitalization by the length of stay for that record. The sum of charges for all hospitalizations was stratified by the 10 expected source of payment categories defined by the Office of Statewide Health Planning and Development (*19*). Hospital charges for Medi-Cal, Medicare, county indigent programs, and other government were combined to estimate the total charge to government payers. The annual sums of charges were adjusted for inflation and standardized to 2011 by using the US Department of Labor Consumer Price Index and were used to assess changes in total charges over time (*28*). All charge figures were calculated in US dollars. When a total charge was indicated as invalid, unknown, or no charge, the records were excluded from analyses. Approximately 8% of hospitalization records had invalid, unknown, or no charge indicated.

### Statistical Analysis

For all, initial, and subsequent hospitalizations and for all primary pulmonary, other forms of progressive, and meningeal coccidioidomycosis hospitalizations, *z* tests were used to compare rates for 2011 with those for 2000. In addition, bivariate relative risks were calculated for the effects of year (2000–2011), sex (female, male), age group (0–19, 20–39, 40–59, and >60 years), and race/ethnicity (White, African American, Hispanic, Other) on patient initial hospitalization. Factors with significant bivariate relative risks were then included in a multivariate negative binomial regression model, which was used to test for statistical significance of the trend in initial hospitalization statewide during 2000–2011. Negative binomial regression models controlling for sex, age group, and race/ethnicity were also used to test for statistical significance of the trends in initial hospitalization in the endemic and less endemic regions. Statistical significance was defined as a p value of <0.05. SAS 9.2 software (SAS Institute Inc., Cary, NC, USA) was used for analyses.

## Results

### Hospitalizations and Coccidioidomycosis Diagnoses

During 2000–2011, there were 25,217 coccidioidomycosis-associated hospitalizations in California; hospitalizations increased from 1,074 in 2000 to 3,197 in 2011 (Table 1). The hospitalization rate per 100,000 population in 2011 was 8.6, a significant increase from the 2000 rate of 3.2/100,000 population (p<0.0001). Of the 25,217 hospitalizations, 15,747 (62%) were initial hospitalizations and 9,470 (38%) were subsequent hospitalizations. Of the initial hospitalizations, 9,568 (61%) were for a primary diagnosis of coccidioidomycosis. The initial hospitalization rate per 100,000 population in 2011 was 5.0, a 2-fold increase from the 2000 rate of 2.3/100,000 population (p<0.0001), and the rate of subsequent hospitalizations in 2011 was 3.6/100,000 population, a >4-fold increase from the 2000 rate of 0.8/100,000 population (p<0.0001).

**Table 1:** Coccidioidomycosis-associated hospitalizations, California, 2000–2011

Variable	No. (%), 2000–2011	No. (rate/100,000 population)	
		2000	2011
Hospitalizations			
All	25,217 (100)	1,074 (3.2)	3,197 (8.6)*
Initial	15,747 (62.4)	798 (2.3)	1,851 (5.0)*
Subsequent	9,470 (37.6)	276 (0.8)	1,346 (3.6)*
Type of coccidioidomycosis†			
Primary pulmonary	12,041 (47.7)	372 (1.1)	1,609 (4.3)*
Other forms of progressive	4,539 (18.0)	224 (0.7)	559 (1.5)*
Meningitis	3,208 (12.7)	195 (0.6)	384 (1.0)*
Pulmonary, unspecified	2,657 (10.5)	124 (0.4)	335 (0.9)
Unspecified	2,372 (9.4)	105 (0.3)	320 (0.9)
Chronic pulmonary	1,159 (4.6)	80 (0.2)	111 (0.3)
Primary extrapulmonary	163 (0.6)	5 (<0.1)	14 (<0.1)

*All hospitalization rates for 2011 were significantly greater than those for 2000 (p<0.0001, z tests). †Numbers, percentages, and rates of all hospitalizations (n = 25,217). Multiple coccidioidomycosis diagnosis codes may be indicated for a single hospitalization.

For all hospitalizations, the most common types of coccidioidomycosis diagnoses were for primary pulmonary (48%), other forms of progressive (18%), and coccidioidal meningitis (13%) (Table 1). The rate of any hospitalization for primary pulmonary coccidioidomycosis was ≈4-fold higher in 2011 than in 2000 (4.3 vs. 1.1 hospitalizations/100,000 population), and the rate of hospitalization for other forms of progressive disease and meningitis was ≈2-fold higher in 2011 than in 2000 (p<0.0001 for all).

Of 15,747 patients initially hospitalized for coccidioidomycosis, 3,824 (24%) were readmitted to a hospital during the study period: 2,004 (52%) were readmitted once, 746 (20%) were readmitted twice, and 1,074 (28%) were readmitted >3 times. The median time from initial hospitalization discharge to first readmission was 47 days (range 0–4,252 days). Of those readmitted, 3,006 (79%) were readmitted within 1 year after the initial hospitalization discharge.

A total of 1,220 (8%) patients hospitalized for coccidioidomycosis died during an initial or subsequent hospitalization. For these patients, there was a diagnosis during the final hospitalization of primary pulmonary coccidioidomycosis for 628 (51%), other forms of progressive disease for 309 (25%), and coccidioidal meningitis for 196 (16%).

### Hospitalizations by Region

During the study period, hospitalized patients were residents of 56 of the 58 California counties. Approximately 50% of patients initially hospitalized for coccidioidomycosis resided in 1 of the 6 endemic region counties. The rate of initial hospitalizations in the endemic region increased nearly 3-fold from 2000 (12.0 initial hospitalizations/100,000 population) through 2011 (34.6 initial hospitalizations/100,000); in the less endemic region, the rate increased 1.6-fold from 2000 (1.6 initial hospitalizations/100,000 population) through 2011 (2.5 initial hospitalizations/100,000 population) (Table 2). In the endemic region, the highest annual hospitalization rates were consistently observed in Kern and Kings Counties.

**Table 2:** Coccidioidomycosis-associated initial hospitalizations by patient residence, California, 2000–2011*

Patient residence†	No. (%), 2000–2011, N = 15,747	No. (rate/100,000 population)	
		2000	2011
Endemic region, county	7,683 (48.8)	281 (12.0)	990 (34.6)
Fresno	1,450 (9.2)	30 (3.7)	171 (18.1)
Kern	4,016 (25.5)	155 (23.3)	544 (61.9)
Kings	760 (4.8)	20 (15.4)	99 (63.5)
Madera	125 (0.8)	5 (4.0)	20 (12.8)
San Luis Obispo	398 (2.5)	23 (9.3)	49 (17.9)
Tulare	934 (5.9)	48 (13.0)	107 (23.6)
Less endemic region	8,064 (51.2)	517 (1.6)	861 (2.5)

*For this study, 6 California counties where coccidioidomycosis is endemic were defined as the endemic region, and all other counties, where coccidioidomycosis is less endemic, were defined as the less endemic region. †For patients admitted from prison or jail, patient residence was based on the location of the prison or jail.

### Hospitalization Trends

**Figure 1:**
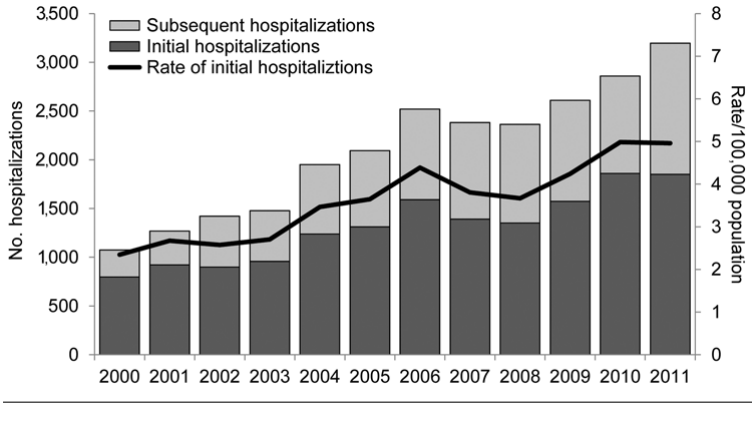
Numbers and annual rates of initial and subsequent coccidioidomycosis-associated hospitalizations (N = 25,217) by year of admission, California, 2000–2011.

**Figure 2:**
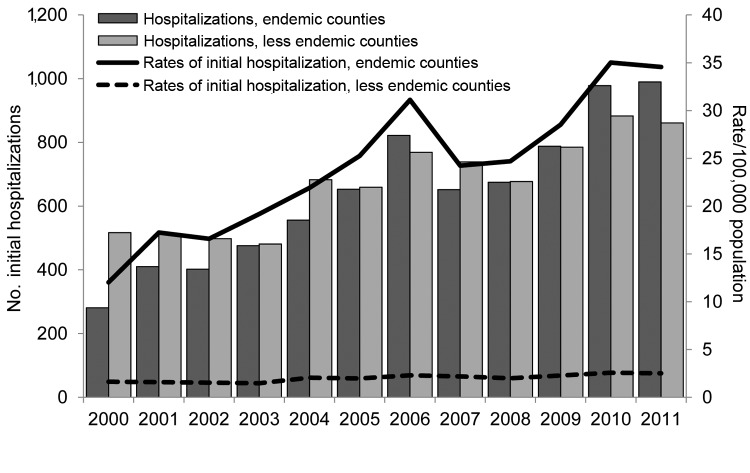
Numbers and annual rates of initial coccidioidomycosis-associated hospitalizations (N = 15,747) in endemic and less endemic regions of California by year of admission, 2000–2011. For this study, 6 California counties (Fresno, Kings, Kern, Madera, San Luis Obispo, and Tulare) where coccidioidomycosis is endemic were defined as the endemic region, and all other counties, where coccidioidomycosis is less endemic, were defined as the less endemic region.

The annual number of hospitalizations increased steadily during 2000–2006, followed by a slight decrease through 2008 and then a steady increase through 2011 (Figure 1). Using multivariate analysis, we adjusted for sex, age group, and race/ethnicity and determined that the increasing trend in initial hospitalizations during 2000–2011 was significant (p<0.0001). Over this period, there was also an overall increase in the annual rates of initial hospitalizations in the endemic and less endemic regions; however, rates in the endemic region were 7–14 times higher than those in the less endemic region (Figure 2). When controlled for sex, age group, and race/ethnicity, trends in initial hospitalizations in the 2 regions increased significantly (p<0.0001) during 2000–2011.

### Demographic Characteristics

During 2000–2011, the overall initial hospitalization rate increased 2-fold; when the analysis was stratified by sex, age group, and race/ethnicity, a similar increase was observed within most strata (Table 3). Most (69%) initial hospitalizations were for men; the annual initial hospitalization rate for men (6.7/100,000 population in 2011) was consistently more than twice that for women (3.2/100,000 population in 2011). In most years, the annual initial hospitalization rates increased steadily by advancing age group and then decreased slightly for patients >80 years of age; the highest 2011 initial hospitalization rate (8.5/100,000 population) was for persons 70–79 years of age. For race/ethnicity, the annual initial hospitalization rates were highest for African Americans (8.9/100,000 population in 2011) and lowest for Asian/Pacific Islanders (2.0/100,000 population in 2011).

**Table 3:** Coccidioidomycosis-associated initial hospitalizations by patient demographic characteristics, California, 2000–2011

Characteristic	No. (%), 2000–2011, N = 15,747	No. (rate/100,000 population)	
		2000, n = 798 (2.3)	2011, n = 1,851 (5.0)
Sex			
F	4,870 (30.9)	256 (1.5)	607 (3.2)
M	10,876 (69.1)	542 (3.2)	1,244 (6.7)
Age, y			
0–9	287 (1.8)	8 (0.2)	38 (0.8)
10–19	680 (4.3)	25 (0.5)	91 (1.7)
20–29	1,630 (10.4)	64 (1.3)	210 (3.7)
30–39	2,476 (15.7)	147 (2.7)	280 (5.4)
40–49	3,312 (21.0)	162 (3.2)	361 (7.0)
50–59	3,190 (20.3)	181 (5.2)	399 (8.3)
60–69	2,032 (12.9)	99 (4.7)	235 (7.2)
70–79	1,375 (8.7)	78 (4.6)	150 (8.5)
>80	765 (4.9)	34 (3.7)	87 (7.2)
Race/ethnicity			
White	6,612 (42.7)	381 (2.4)	697 (4.8)
African American	1,886 (12.2)	106 (4.8)	190 (8.9)
Hispanic	5,469 (35.3)	233 (2.1)	747 (5.2)
Native American/Alaska Native	66 (0.4)	3 (1.7)	10 (6.5)
Asian/Pacific Islander	1,033 (6.7)	52 (1.3)	104 (2.0)
Other	425 (2.7)	12 (1.9)	66 (7.2)

Multivariate negative binomial regression analysis showed that male sex, older age group, and African American and Hispanic race/ethnicities were significantly associated with an increased risk for initial hospitalization (p<0.0001 for all) (Table 4). This increased risk was 2.48 times higher for men than women. The risk for initial hospitalization for African Americans and Hispanics was 2.09 and 1.31 times higher, respectively, than that for Whites. The risks for initial hospitalization for persons 20–39, 40–59, and >60 years of age were 4.22, 7.73, and 9.50 times higher, respectively, than that for persons 0–19 years of age (Table 4).

**Table 4:** Risk for coccidioidomycosis-associated initial hospitalization, by various demographic characteristics, California, 2000–2011*

Characteristic	Multivariate RR (95% CI)†
Year	1.06 (1.06–1.07)
Sex	
F	Referent
M	2.48 (2.33–2.63)
Age, y	
0–19	Referent
20–39	4.22 (3.82–4.66)
40–59	7.73 (7.01–8.52)
>60	9.50 (8.59–10.50)
Race/ethnicity	
White	Referent
African American	2.09 (1.92–2.28)
Hispanic	1.31 (1.21–1.41)
Other‡	0.83 (0.76–0.90)

*N = 15,747. RR, relative risk. †RRs were calculated by using multivariate negative binomial regression. All p values were <0.0001. ‡Includes persons of Asian/Pacific Islander, Native American/Alaska Native, and other race/ethnicity.

A total of 1,374 (8.7%) patients initially hospitalized with coccidioidomycosis were admitted from a prison or jail. Of those, 1,103 (80%) were admitted from prisons or jails in the endemic region of California. Thirty-eight percent of hospitalized persons from Kings County were initially admitted from prison or jail, compared with 25% of persons from Fresno County and 10% from Kern County. The median age of persons admitted from prison or jail was 42 years (range 18–88), and 99.5% of these patients were men. Of the 1,374 patients initially admitted to the hospital from prison or jail, 342 (25%) were White, 379 (28%) were African American, and 419 (30%) were Hispanic. The number of initial hospital admissions for persons in prison or jail increased from 28 in 2000 to 201 in 2011. When initial hospitalizations for persons in prison or jail were excluded from the multivariate analysis, the risk for initial hospitalization for men, persons in older age groups, and persons of African American or Hispanic race/ethnicity remained significantly higher than the risk for the reference group (p<0.05 for all) (data not shown).

Records for 33% (5,176) of the coccidioidomycosis-related initial hospitalizations had a diagnosis code indicating a concurrent condition that could increase the risk for infection or severe disease. Of those, 34% (1,760) had diagnosis codes for immunocompromising conditions. Approximately 2.8% (439) of initial hospitalizations had a diagnosis code of HIV infection or AIDS, compared with an estimated 0.3% of the California population. Approximately 25% (3,786) of adults initially hospitalized for coccidioidomycosis had a diagnosis of diabetes. When our data and the CHIS data were age-adjusted to the 2000 US standard population, 22% had a diagnosis of diabetes, compared with an age-adjusted 7.7% of the general California population. Twelve percent (185) of women 18–45 years of age had a diagnosis code indicating pregnancy, compared with the state average of 4% from CHIS.

### Length of Stay and Hospital Charges

During 2000–2011, the median length of stay per hospitalization was 6 days; this length of stay did not vary substantially over time. For ≈52% of those hospitalized, the total per person length of stay was >1 week. The median charge per day was ≈$6,800 US, and the median total hospital charge per patient was US $55,062 (range ≈$1,000 to >$6 million).

During 2000–2011, the total charges for all coccidioidomycosis-associated hospitalizations in California was US $2.2 billion, and the average annual total was US $186 million (Table 5). After we adjusted for inflation, the annual total charges increased from US $73 million in 2000 to US $308 million in 2011. For the expected source of payment category, private coverage had the highest total hospital charges during the study period (total US $713 million; average annual total US $59 million). Government payers (defined as Medi-Cal, Medicare, other government, and county indigent payers) were the expected source of payment for 62% of charges. For all government payers combined, the total (US $1.4 billion) and average annual total (US $115 million) of hospital charges were each nearly twice those for private coverage (Table 5).

**Table 5:** Total and average annual charges, by expected source of payment category, for coccidioidomycosis-associated hospitalizations, California, 2000–2011*

Payment category	% Total categories	Total, US $	Average annual total, US $
Private coverage	32	713,390,109	59,449,176
Government	62	1,388,671,670	115,722,639
Medi-Cal	27	595,837,721	49,653,143
Medicare	25	567,965,499	47,330,458
Other government	7	161,878,874	13,489,906
County indigent programs	3	62,989,577	5,249,131
Self-pay	4	92,892,777	7,741,065
Workers compensation	1	18,209,024	1,517,419
Other payer	1	11,775,902	981,325
Other indigent	<1	7,553,126	629,427
Invalid/unknown	<1	593,365	118,673
Total	100	2,233,085,973	186,159,724

*Unknown, invalid, and missing charges were excluded from this analysis. Charity care charges coded as $1 were also excluded. Approximately 8% of hospitalizations were missing total charge data. These charges are not adjusted for inflation.

## Discussion

The number of initial and subsequent coccidioidomycosis-associated hospitalizations in California increased substantially during 2000–2011, totaling >$2 billion US in hospital charges, most of which was covered by government funding. Rates for initial hospitalizations were substantially higher for men than women, for African Americans and Hispanics than Whites, for persons of older age than younger age, and for persons residing in endemic region counties than in less endemic region counties.

Our study documents a large financial cost related to coccidioidomycosis-associated hospitalizations in California, yet our estimates do not include indirect costs linked to the hospitalizations (e.g., costs related to child care, missed days of work, and follow-up outpatient care). These costs are likely substantial because >50% of patients were hospitalized for >1 week. In addition, hospitalization charges are only a portion of the total financial cost of coccidioidomycosis because most patients do not require hospitalization but may accrue substantial costs during outpatient care (*9*).

The increases in coccidioidomycosis-associated hospitalizations in California were observed in the endemic and less endemic regions. Nearly 50% of initial hospitalizations were for persons residing in the endemic region counties, even though only 7% of the California population lived in these counties during 2000–2011. The increase in hospitalizations is consistent with the dramatic increase in the number of reported coccidioidomycosis cases in California, yet the reasons for the increase are unclear (*6*,*12*–*15*). Contributing factors may include changes in climate and rainfall patterns, leading to proliferation of *C. immitis* fungi in the soil; soil-disturbing construction activities; an increase in susceptible persons moving to disease-endemic areas; and heightened awareness and diagnosis (*1*,*2*,*5*,*16*,*29*).

The high initial hospitalization rate for men in our study is consistent with findings from previous research. This finding may reflect the higher risk for men than women of developing primary pulmonary and extrapulmonary disease once infected, and it may reflect an increased risk for infection among men because of dust exposure in male-dominated occupations, such as construction and farm work (*7*,*8*,*30*,*31*). The high initial hospitalization rates observed for African American and Hispanic residents may be associated with the well-known increased risk for disseminated disease in African Americans and with a reported increased risk for symptomatic disease for both of these racial/ethnic groups in California (*6*,*7*,*32*). Hector et al. (*6*) reported that of the coccidioidomycosis cases reported in California during 2001–2009 with complete race/ethnicity data, a higher proportion were among persons of African American and Hispanic race/ethnicity than would be expected on the basis of the proportion of these racial/ethnic groups in the population. A contributing factor to this finding may be the large populations of Hispanics living and working in the endemic region counties of California. However, the principle causes of the disproportionate race/ethnicity-associated risk for coccidioidomycosis is not well understood and may be attributable to variations in genetic susceptibility (*6*,*7*,*32*).

The percentages of coccidioidomycosis-infected persons hospitalized with HIV infection or AIDS, diabetes, and pregnancy were greater than estimated population percentages, and these conditions are known to increase the risk for developing severe pulmonary or disseminated coccidioidomycosis (*7*,*33*). It is possible, however, that these concurrent conditions put persons at risk for hospitalization in general and that some hospitalizations were primarily for the concurrent condition rather than coccidioidomycosis. An increased risk for severe disease associated with concurrent conditions may also explain the higher rate of initial hospitalization that we observed for persons in older age groups; it has been reported that decreasing immunity in the elderly is correlated with the presence of concurrent conditions (*33*,*34*). In addition, the decline in immunity in elderly persons particularly affects cell-mediated immunity, which is vital for protection against coccidioidomycosis (*33*,*35*).

Almost 9% of patients initially hospitalized for coccidioidomycosis were admitted from prison or jail. A high proportion of these patients were nonwhite men, a finding that reflects the general California inmate population (*36*). Of the 33 California correctional and rehabilitation facilities for adults, 11 are located in the endemic region, and most patients in this study who were hospitalized from prison or jail were admitted from facilities in the endemic region. However, many prisoners in facilities in the endemic region may have resided in or been transferred from counties in the less endemic region, and immunity to coccidioidomycosis would have been less likely to have developed in persons from the less endemic region. Despite the demographic differences between patients admitted to the hospital from prison or jail and those admitted from the general population, the results of the multivariate analyses did not change substantially when data for prison- or jail-admitted hospitalizations were included.

There were several limitations in this study. Some hospitalizations with a secondary diagnosis code for coccidioidomycosis could have been for a condition unrelated to coccidioidomycosis, and inclusion of such hospitalizations in the analyses could have led to an overestimation of the incidence of disease. Some readmissions (e.g., some occurring >1 year after discharge for the initial hospitalization) might also have been for an unrelated medical issue. However, most of the initial hospitalizations had a primary diagnosis of coccidioidomycosis, and most first readmissions occurred <1 year after initial discharge. Some hospitalizations in this study may have also been misclassified as initial hospitalizations because data were not reviewed to identify coccidioidomycosis-associated hospitalizations that occurred before January 1, 2000. Such a misclassification would have led to an overestimation of the number of initial hospitalizations occurring in the earlier years more than in the later years; if that was the case, it would indicate that the increasing trend found in initial hospitalizations may be even greater. The estimates of total charges likely underestimated the financial cost of coccidioidomycosis-associated hospitalizations because charge data were missing for >8% of the hospitalizations. In addition, the California Patient Discharge Data Set did not collect data from federal hospitals in California, a fact that would lead to an underestimation of hospitalization and hospital charge figures.

The increasing health and financial toll of coccidioidomycosis-associated hospitalizations in California are a major public health challenge. Efforts are needed to reduce the incidence of disease, yet options for the prevention of coccidioidomycosis are limited. Although a vaccine is not currently available, vaccine research is under way (*37*). Early diagnosis, close follow up, and appropriate treatment of patients at risk for severe or disseminated disease may decrease the number of long-term illnesses and deaths. Thus, efforts should be made to increase disease awareness and promote early recognition among health care providers and the public. In addition, prevention messages on how to minimize or avoid breathing in dusty air should be communicated more widely to persons living in or traveling to areas where *Coccidioides* fungi are endemic, particularly to persons at risk for severe disease and hospitalization.
